# Root Antioxidant Mechanisms in Relation to Root Thermotolerance in Perennial Grass Species Contrasting in Heat Tolerance

**DOI:** 10.1371/journal.pone.0138268

**Published:** 2015-09-18

**Authors:** Yi Xu, Patrick Burgess, Bingru Huang

**Affiliations:** Department of Plant Biology and Pathology, Rutgers University, New Brunswick, NJ, 08901, United States of America; Chinese Academy of Sciences, CHINA

## Abstract

Mechanisms of plant root tolerance to high temperatures through antioxidant defense are not well understood. The objective of this study was to investigate whether superior root thermotolerance of heat-tolerant *Agrostis scabra* relative to its congeneric heat-sensitive *Agrostis stolonifera* was associated with differential accumulation of reactive oxygen species and antioxidant scavenging systems. *A*. *scabra* ‘NTAS’ and *A*. *stolonifera* ‘Penncross’ plants were exposed to heat stress (35/30°C, day/night) in growth chambers for 24 d. Superoxide (O_2_^-^) content increased in both *A*. *stolonifera* and *A*. *scabra* roots under heat stress but to a far lesser extent in *A*. *scabra* than in *A*. *stolonifera*. Hydrogen peroxide (H_2_O_2_) content increased significantly in *A*. *stolonifera* roots but not in *A*. *scabra* roots responding to heat stress. The content of antioxidant compounds (ascorbate and glutathione) did not differ between *A*. *stolonifera* and *A*. *scabra* under heat stress. Enzymatic activity of superoxide dismutase was less suppressed in *A*. *scabra* than that in *A*. *stolonifera* under heat stress, while peroxidase and catalase were more induced in *A*. *scabra* than in *A*. *stolonifera*. Similarly, their encoded transcript levels were either less suppressed, or more induced in *A*. *scabra* roots than those in *A*. *stolonifera* during heat stress. Roots of *A*. *scabra* exhibited greater alternative respiration rate and lower cytochrome respiration rate under heat stress, which was associated with suppression of O_2_^-^ and H_2_O_2_ production as shown by respiration inhibitors. Superior root thermotolerance of *A*. *scabra* was related to decreases in H_2_O_2_ and O_2_^-^ accumulation facilitated by active enzymatic antioxidant defense systems and the maintenance of alternative respiration, alleviating cellular damages by heat-induced oxidative stress.

## Introduction

Heat stress is a major abiotic factor which limits plant growth and productivity, particularly in cool-season (C_3_) species. Plants undergo various physiological and cellular changes as heat stress progresses including oxidative damage caused by production of reactive oxygen species (ROS), namely hydrogen peroxide (H_2_O_2_) and superoxide (O_2_^-^) [1–4]. ROS are typically produced when electrons seeking a lower energy state are transferred to molecular oxygen during inhibition of carbon-fixation in leaves or elevated cytochrome respiration in leaves and roots, among other metabolic processes [5]. Although many of them are important signaling molecules in the regulation of plant growth, overproduction and accumulation of ROS in various plant organs decreases cellular membrane stability leading to oxidative damages of nucleic acid, lipids, and proteins [6–9].Therefore, methods which reduce oxidative damages by limiting production or accumulation of ROS are critical for improving plant tolerance or adaptation to heat stress.

Plant adaptation to oxidative stress may be aided in part by non-enzymatic and enzymatic ROS scavenging systems [6]. Non-enzymatic compounds include glutathione (GSH) and ascorbate (ASA) which possess intrinsic antioxidant properties and serve as electron donors to reduce ROS accumulation[10].There are two distinct antioxidant enzymatic pathways in plants, the first of which utilizes superoxide dismutase (SOD), catalase (CAT), and/or peroxidase (POD) located subcellularly in mitochondria, chloroplasts, and peroxisomes [11–13]. SOD converts O_2_^-^ to less-damaging H_2_O_2_ which is subsequently split into non-damaging water and oxygen by POD or CAT. The second antioxidant enzymatic pathway utilizes ascorbate peroxidase (APX), glutathione reductase (GR), monodehydroascorbate reductase (MR), and dehydroascorbate reductase (DR)to reduce H_2_O_2_ to water and oxygen by controlling the balance of GSH and ASA in plant leaves [11, 12]. Non-enzymatic and enzymatic pathways serve important roles for antioxidant metabolism enhancing plant tolerance to various stresses, though the specific compounds or enzymes contributing to stress defense vary across plant species, varieties, age, organs, and in response to stress type, severity, and duration [9, 14–18]. Most of previous work reported leaf antioxidant mechanisms in relation to stress defense, but limited information is available on how roots may survive high temperature through antioxidant defense.

It is well documented that roots are more sensitive than shoots under heat though the specific mechanisms underlying root susceptibility to heat stress or thermotolerance to tolerate high temperatures are not well understood [4, 19]. In addition, ROS scavenging capacity may differ between roots and shoots due the variations in their subcellular locations in different tissues. ROS is produced and subsequently scavenged in chloroplasts, mitochondria, and peroxisome in leaves, whereas it is mainly generated in mitochondria for root tissues [20–22]. It remains unknown whether the antioxidant scavenging components differ between the two organ types and how individual components affect root thermotolerance. Specifically in plant roots of various species, mitochondrial H_2_O_2_ and O_2_^-^increase as root respiration increases during prolonged heat stress [23–25]. There are two distinct pathways by which plant mitochondria carry out respiratory electron transport, the cytochrome pathway which accelerates ROS production and the alternative pathway which slows production rates and reduces net accumulation of ROS [8, 26]. The cytochrome pathway is characterized by electron transfer governing ATP synthesis at the expense of ROS production due to the ubisemiquinone radical which transfers an electron to oxygen prompting superoxide formation [27]. The alternative pathway is characterized by enhanced activity of alternative oxidase (AOX) which effectively diverts high-energy electrons produced by over-reduction of cytochrome oxidase and avoids significant ROS production [28, 29]. Nitric oxide (NO) has been shown to induce alternative respiration by inhibiting cytochrome oxidase activity and is also a complementary partner to ROS for determining cell fate or for signaling responses during stress onset [30–33]. Utilizing NO to prompt a shift towards alternative respiratory pathways and significantly enhance ROS scavenging in various plant organs has not been fully understood and deserves further attention.

Thermal bentgrass (*Agrostis scabra*) thrives alongside geothermal vents with soil temperature exceeding 40°C in Yellowstone National Park, USA and roots can actively grow under extremely high soil temperatures [34] while many of its congeneric species, such as *A*. *stolonifera*, a commonly used turf and forage grass species, are very sensitive to heat stress and majority of roots lost viability as temperature increases above 25°C [19]. The superior root thermotolerance of thermal *A*. *scabra* has been attributed to various mechanisms facilitating respiratory acclimation, changes in carbon allocation, cellular membrane stability, and activation of stress defense proteins such as chaperones [19, 35–39].However, many questions remain unanswered regarding the interrelationship of ROS, enzymatic and non-enzymatic antioxidants, and respiratory pathways collectively governing root thermotolerance in cool-season grasses. Comparative analysis between thermal *A*. *scabra* and heat-sensitive *A*. *stolonifera* will contribute to a better understanding of heat tolerance mechanisms and may aid in future breeding selections of heat-tolerant germplasm in cool-season grasses. Therefore, the objective of this study were to determine whether root thermotolerance was due to activation of non-enzymatic antioxidant production or antioxidant enzymes at both enzymatic activity and gene expression levels and investigate the relationship of root respiration and ROS production in relation to root thermotolerance in thermal *A*. *scabra* compared to heat-sensitive *A*. *stolonifera* during prolonged heat stress.

## Materials and Methods

### Plant materials and growth conditions

Tillers (30 per individual plant) of *A*. *stolonifera* ‘Penncross’ or *A*. *scabra* ‘NTAS’ were harvested from stock plants and transferred to plastic containers(57 x 44 x 30 cm, 12 drainage holes) filled with fritted clay medium (Profile Products, Deerfield, IL). Plants were established for 35 d in a greenhouse set to 23/20°C (day/night), 60% relative humidity (RH), 14 h photoperiod, and 500 μmol m^-2^ s^-1^ photosynthetically active radiation (PAR) from natural sunlight and supplemental lighting. Plants were irrigated daily, fertilized twice per week with half-strength Hoagland’s nutrient solution [40], and trimmed to 2 cm once per week during establishment. Plants were not trimmed during the final week of establishment to allow for sufficient regrowth prior to stress imposition, after which time all plants were transferred to controlled-environment growth chambers (Environmental Growth Chamber, Chagrin Falls, Ohio, USA).

### Treatments and experimental design

Plants were maintained in controlled-climate growth chambers set to 22/18°C (day/night), 600 μmol m^-2^ s^-1^ PAR, 60% RH, and 14 h photoperiod for one week prior to stress imposition, after which time air temperature was raised to 35/30°C to impose heat stress for 24 d. During stress treatment, plants were irrigated daily, fertilized twice per week with half-strength Hoagland’s nutrient solution and not trimmed. The experiment was arranged in a split-plot design with temperature treatment (control or heat) as the main plot and grass species (*A*. *scabra* or *A*. *stolonifera*) as subplots. Each treatment was replicated in four containers and each container included four individual plants per species. Since each chamber was only able to accommodate two containers, a relocation strategy was introduced between four containers in their respective chambers every 3 d to avoid possible confounding effects of unique chamber environmental variations from occurring.

### Root physiological analysis

Following 24 d stress treatment, roots were washed free of fritted clay and a subset from each plant was collected to quantify root electrolyte leakage (EL) and malondialdehyde (MDA) content. EL was measured according to the procedure described by Blum and Ebercon [41] and used to indicate the cellular membrane stability or membrane status following treatment. A subset of roots was rinsed with deionized water to remove exogenous solutes and placed in a test tube containing 30 mL deionized water. Tubes were agitated in a conical flask shaker for 12 h and the initial conductance (C_i_) of incubation solution measured using a conductivity meter (YSI Model 32, Yellow Springs, OH). Tubes containing root tissue were then autoclaved at 121°C for 20 min and again agitated for 12 h. The maximal conductance (C_max_) of incubation solution was then measured and EL (%) was calculated as ((C_i_/C_max_) × 100).

MDA is the final product of lipid peroxidation in plant tissue and was measured according to the procedure described by Zhang and Kirkham [17] with slight modifications. A subset of roots (0.5 g) was homogenized in 6 mL 0.1% trichloroacetic acid (TCA) and the homogenate was centrifuged at 10,000 g for 10 min. 1mL supernatant was added to 4 mL 10% TCA containing 0.5% thiobarbituric acid (TBA). The mixture was incubated at 95°C for 30 min, quickly cooled on ice, and centrifuged at 10,000 g for 10 min at 4°C. The absorbance of supernatant was measured at 532 and 600 nm using a spectrophotometer (Spectronic Instruments, Rochester, NY). The concentration of MDA was calculated using MDA’s extinction coefficient of 155 mM^-1^ cm^-1^[42]. All content levels were expressed as the mean (the average content in g fresh weight (FW)) ± SE (the standard error) of four biological replicates.

### Histochemical staining for presence of hydrogen peroxide and superoxide

Histochemical staining for the presence of hydrogen peroxide and superoxide was performed following 24 d stress treatment, using procedures described in Thordal-Christensen et al. [43] and Dunand et al. [44], with slight modifications respectively. To evaluate the presence of hydrogen peroxide (H_2_O_2_), a subset of roots was stained with 1% (w/v) 3-diaminobenzinidine (DAB; pH 3.8) for 2 h and subsequently rinsed with deionized water. To evaluate the presence of superoxide (O_2_^-^), a subset of roots was stained with2 mM nitroblue tetrozolium (NBT) in 20 mM phosphate-buffered saline (PBS; pH 6.8) for 30 min and subsequently rinsed with deionized water. DAB or NBT-stained roots were observed visually using an Olympus FSX100 Bio-imaging navigator (Central Valley, PA) and pictures were captured using bright-field single-shot mode at 4.2x magnification.

### Quantification of reactive oxygen species and antioxidant compounds

Superoxide (O_2_^-^) production rate was measured according to the procedure described by Bian and Jiang [15] with slight modifications. Root tissue (0.5 g)was ground to a powder in liquid nitrogen, homogenized in 1 mL 50 mM Tris-HCl (pH 7.5), and centrifuged at 5,000 g for 10 min at 4°C. 200 μL supernatant was added to 800 μL 0.5 mM 3-bis(2-methoxy-4-nitro-5-sulfophenyl)-2H-tetrozolium-5-carboxanilide inner salt (XTT). XTT reduction was recorded once per minute for 3 min at 470 nm using a spectrophotometer (Spectronic Instruments, Rochester, NY) and the background absorbance was corrected with 50 units of superoxide dismutase (SOD). The O_2_^-^ production rate was calculated using 2.16 ×10^4^ M^-1^ cm^-1^ extinction coefficient and expressed as μmol O_2_^-^ min^-1^ g^-1^ FW [45].

Hydrogen peroxide (H_2_O_2_) content was measured according to the procedure described by Zhou et al. [46] with slight modifications. Root tissue (0.5 g) was homogenized in 5 mL 5% (w/v) TCA and the homogenate was centrifuged at 10,000 g for 20 min at 4°C. The supernatant was adjusted to pH 8.4 with 17 M ammonia solution, briefly centrifuged to remove large particles, and divided into 1 mL aliquots. 8 μg catalase was added to one aliquot to serve as the blank and not added to other aliquots. 1 mL of colorimetric reagent solution containing 10 mg 4-aminoantipyrine, 10 mg phenol, and 5 mg peroxidase in 100 mM acetic acid buffer (pH 5.6) was added to each aliquot and the color reaction was incubated for 10 min at 30°C. Following incubation, the absorbance was measured at 505 nm using a spectrophotometer (Spectronic Instruments, Rochester, NY) and H_2_O_2_ content determined based on standard curve generated with known H_2_O_2_concentrations.All content levels were expressed as the mean (the average content in g FW) ± SE of four biological replicates.

### Quantification of non-enzymatic antioxidant content

Glutathione (GSH) and ascorbate (ASA) contents were measured according to the procedure described by Guri [47] with slight modifications. Root tissue (0.5 g) was homogenized in 5 mL 5% (w/v) TCA on ice and centrifuged at 16,000 g for 20 min at 4°C. The homogenate was titrated to a pH range of 6–8 with 1.5 mL 0.1 M NaOH. 2 mL titrated homogenate was added to0.5 mL 0.2 M sodium phosphate buffer (pH 7.0), 0.4 mL deionized water, and 0.1 mL 0.5% (w/v) dithiobis-2-nitrobenzoic acid (DTNB) and absorbance measured at 412 nm using a spectrophotometer (Spectronic Instruments, Rochester, NY). Titrated homogenate containing sodium phosphate buffer, deionized water, but lacking DTNB served as the blank. GSH and ASA content were determined based on standard curves generated with known concentrations of each compound.

Reduced and total ASA content were measured according to the procedure described by Ma et al. [48] with slight modifications. Root tissue (0.5 g) was homogenized in 8 ml 5% (w/v) TCA on ice, centrifuged at 10,000 g for 10 min at 4°C, and resulting supernatant used immediately for analysis. For total ASA quantification, the supernatant was incubated in 200 mM sodium phosphate buffer (pH 7.4) and 1.5 mM dithiothreitol (DTT) for 50 min to reduce all oxidized ascorbate (DHA) to ASA. Following incubation, 200 μL 0.5% (w/v) N-ethylmaleimide (NEM) was added to remove excess DTT. The resulting solution (0.8 ml)was then added to a reaction mixture containing 1 mL 10% (w/v) TCA, 800 μL 42% (w/v) o-phosphoric acid, 800 μL 65 mM 2,2’-dipyridyl in 70% (v/v) ethanol, and 400 μL 3% (w/v) iron (III) chloride. The reaction was incubated at 42°C for 1 h, and absorbance measured at 525 nm using a spectrophotometer (Spectronic Instruments, Rochester, NY).Reduced ASA was measured using the procedure described above with DTT and NEM substituted with 400 μL deionized water. Reduced and total ASA content were determined based on standard curves generated with known ASA concentrations. All content levels were expressed as the mean (the average content in g FW) ± SE of four biological replicates.

### Quantification of enzymatic activity

Enzyme activity of CAT, POD, SOD, APX, DR, MR, and GR was measured according to the procedures described by Zhang and Kirkham [17]. For each CAT, POD, and SOD assay, 0.5 g root tissue was homogenized in 6 ml 50 mM sodium phosphate buffer (pH 7.0) containing 0.2 mM ethylenediaminetetraacetic acid (EDTA) and 1% (w/v) polyvinylpyrrolidone (PVP) on ice and the homogenates were centrifuged at 15,000 g for 20 min at 4°C. The absorbance were measured at 240, 470, and 560 nm for CAT, POD, and SOD, respectively, using a spectrophotometer (Spectronic Instruments, Rochester, NY).For each APX, DR, MR, and GR assay, 0.5 g root tissue was homogenized in 6 ml 25 mM sodium phosphate buffer (pH 7.8) containing 0.2 mM EDTA and 1% (w/v) PVP and the homogenates were centrifuged at 15,000 g for 20 min at 4°C. Absorbance were measured at 290, 265, 340, and 340 nm for APX, DR, MR, and GR, respectively, using a spectrophotometer (Spectronic Instruments, Rochester, NY). All enzymatic activity levels were expressed as the mean (the average content in mg of protein) x SE of four biological replicates.

### Gene expression analysis of enzymatic antioxidants

Gene expression analysis was performed by quantitative reverse transcriptase polymerase chain reaction (qRT-PCR).Total RNA was isolated from root tissue using TRIzol reagent (Life Technologies, Grand Island, NY) and treated with DNase (TURBO DNA-free kit; Life Technologies, Grand Island, NY) to remove contaminating genomic DNA. 2 μg total RNA was reverse-transcribed using a high-capacity cDNA reverse transcription kit (Life Technologies, Grand Island, NY) and the synthesized cDNA was amplified in a StepOnePlus Real-Time PCR system (Life Technologies, Grand Island, NY) using the following parameters: pre-heat cycle of 95°C for 3 min, 40 cycles of 95°C denaturation for 30 sec, and 60°C annealing/extension for 60 sec. Power SYBR Green PCR Master Mix (Life Technologies, Grand Island, NY) was the intercalating dye used to detect gene expression level. Gene name, accession number, forward and reverse primer sequences are provided in Table 1. A melting curve analysis was performed for each primer pair to confirm its specificity. *Actin2* was used as the reference gene, since its expression was constant throughout treatments. A ΔΔCt method was used to calculate the relative expression level between genes of interest and reference gene, respectively. All transcript levels were expressed as the mean (the average relative expression level) ± SE of four biological replicates.

**Table 1:** Primer sequences of ROS scavenging genes used in qRT-PCR. Proposed gene names, GenBank accession numbers, best BLAST hit names, E-values, sequence identity scores are also listed.

Gene	Accession number	Best BLAST hit	E-value	Identity	Primer sequence	
**CuZn-SOD**	DV867103	JQ269675.1 (*Triticum aestivum*)	3e-161	87%	Forward	CACTGGACCTCACTTCAAC
					Reverse	GTAGCAACACCATCCACTC
**POD2**	DV867327	XM_010230345.1 (*Brachypodium distachyon*)	6e-153	89%	Forward	CTTCGACAACGCCTACTAC
					Reverse	TTTGCCCATGTTCACCA
**CAT1**	DY543619	AJ634002.1 (*Schedonorus arundinaceus*)	0	93%	Forward	TTGCCAATAAGAGGGAGAATG
					Reverse	CGAAGCCGAGCATGTAAG
**APX2**	GR281667	KP852178.1 (*Beckmannia syzigachne*)	0	94%	Forward	AGGACATTGTTGCCCTTTC
					Reverse	GCTCCGTGAAGTAAGAGTTG
**GR**	AB277097	AB277097 (*Hordeum vulgare*)	0	100%	Forward	GATGGAGGCTACTTGCTTTG
					Reverse	GCTAAGACCCACGACAGATA
**MR**	DV865077	KC884831.1 (*Triticum aestivum*)	5e-160	90%	Forward	CCATGAAGCTCTACAACGAG
					Reverse	GTAGAAGTAGGGCAGGTAGT
**DR**	DV853556	HM125046.1 (*Puccinellia tenuiflora*)	0	90%	Forward	GAAAGGTGCCTGTGTTTAATG
					Reverse	GTGATGGAGTTGGGTACTTC
**ACT2**	DY543529	XM_003578821.2 (*Brachypodium distachyon*)	0	93%	Forward	CCTTTTCCAGCCATCTTTCA
					Reverse	GAGGTCCTTCCTGATATCCA

### Quantification of root respiration rate

Root respiration rate was measured according to the procedure described by Rachmilevitch et al. [35] with slight modifications. A subset of roots was detached from shoots, washed free of fritted clay, and immediately transferred into 500 ml Buchner flasks containing 400 ml half-strength Hoagland’s nutrient solution. The nutrient solution contained either 200 μM sodium nitroprusside (SNP) to inhibit the cytochrome respiratory pathway or 10 mM salicylhydroxamic acid (SHAM) to inhibit the alternative respiratory pathway. Solutions containing SNP or SHAM were maintained as an open-flow system by aerating with circulating pumps (Apollo Enterprises Inc., Oxnard, CA) for 30 min, after which time a closed-flow system was created by connecting the terminal air tube to the circulating pump inlet. Vacuum grease and Teflon tape were used to maintain an air-tight seal around the rubber stoppers. CO_2_ evolution rate was measured every 30 min for 2 h by extracting 1 ml air samples from the flasks using air-tight syringes and re-sealable septa affixed to flask side arms. Air samples were then injected into a Shimazu GC-8AIT gas chromatograph (Shimazu, Kyoto, Japan) equipped with a thermal conductivity detector and a stainless steel column (length: 6 ft; I.D.: 0.085”; O.D.: 1/8”) packed with Porapack Q (80/100 mesh).The temperatures for injector, column, and detector were set at 30°C, 150°C, and 150°C, respectively. Helium was used as a carrier gas at a flow rate of 30 mLminute^-1^. Remaining root tissue was dried in an oven at 80°C for 72 h and subsequently weighed on a mass balance. Root respiration rates were expressed as O_2_ uptake rate (mmol h^-1^ g^-1^root dry weight, DW) converted proportionally from CO_2_ evolution rate [34].

### Statistical analysis

Temperature treatment effects and species variations were tested with the analysis of variance using the general linear model in a statistical program (SAS9.0, Cary, NC). Differences in mean values between treatments and between species were determined using the student’s t-test. A P-value of ≤0.05 was considered as statistically significant.

## Results

### Physiological responses for differential root thermotolerance of *A*. *scabra* and *A*. *stolonifera*

**Fig 1:**
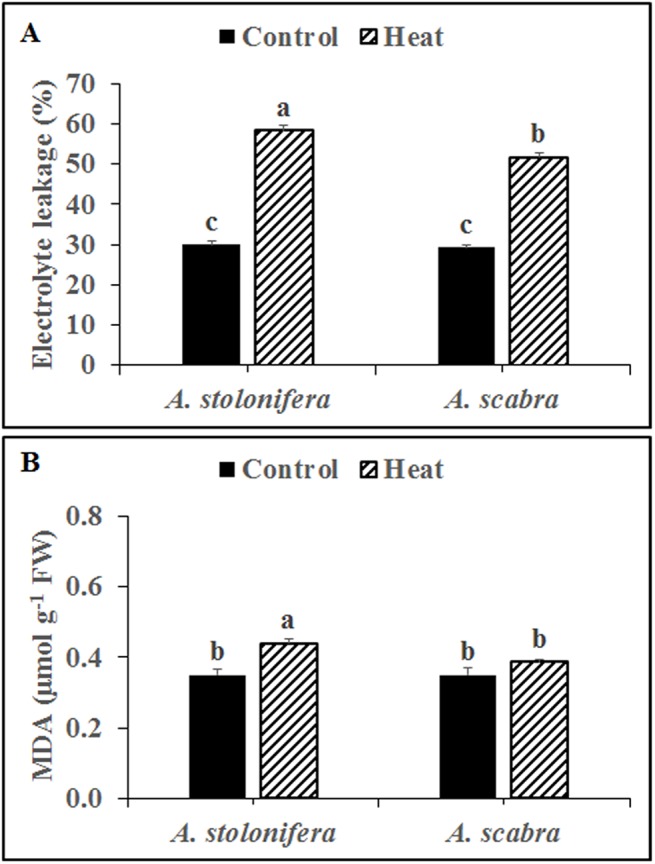
Root electrolyte leakage (A) and MDA content (B) of *A*. *stolonifera* and *A*. *scabra* following control and heat stress treatment. Data shown are the mean ± SE of four biological replicates. Different letters atop bars indicate significant differences exist at the P ≤ 0.05 level.

Heat stress treatment significantly increased root EL for both grass species compared to respective non-stress controls (Fig 1A). EL increased by 95 and 76% for *A*. *stolonifera* and *A*. *scabra*, respectively, due to heat stress. EL did not differ between *A*. *stolonifera* and *A*. *scabra* under non-stress conditions whereas it remained significantly lower (11% decrease) in *A*. *scabra* compared to *A*. *stolonifera* following heat stress treatment. Heat stress treatment significantly increased root MDA content for *A*. *stolonifera* but not *A*. *scabra* compared to respective non-stress controls (Fig 1B). Root MDA content increased by 26 and 11% for *A*. *stolonifera* and *A*. *scabra*, respectively, due to heat stress. MDA content did not differ between *A*. *stolonifera* and *A*. *scabra* under non-stress conditions whereas it remained significantly lower (12% decrease) in *A*. *scabra* compared to *A*. *stolonifera* following heat stress treatment.

### ROS production for differential root thermotolerance of *A*. *scabra* and *A*. *stolonifera*

**Fig 2:**
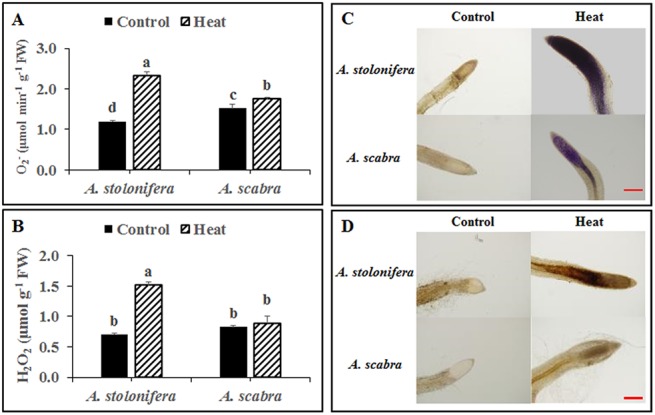
Quantification of O2- (A) and H_2_O_2_ (B) content of *A*. *stolonifera* and *A*. *scabra* following control or heat-stress treatment and histochemical staining of *A*. *stolonifera* and *A*. *scabra* roots with NBT (C) or DAB (D). Data shown are the mean ± SE of four biological replicates. Different letters atop bars indicate significant differences exist at the P ≤ 0.05 level. Bar represents 100 μm.

Heat stress treatment significantly increased root superoxide (O_2_^-^) content for both grass species compared to respective non-stress controls (Fig 2A). Superoxide (O_2_^-^) content increased by 94 and 15% for *A*. *stolonifera* and *A*. *scabra*, respectively, due to heat stress. O_2_^-^ content was significantly higher (27% increase) in *A*. *scabra* than *A*. *stolonifera* under non-stress conditions whereas it remained significantly lower (25% decrease) in *A*. *scabra* compared to *A*. *stolonifera* following heat stress treatment. Heat stress treatment significantly increased root hydrogen peroxide H_2_O_2_ content in *A*. *stolonifera* but not in *A*. *scabra* compared to respective non-stress controls (Fig 2B). H_2_O_2_ content increased by 117 and 5% for *A*. *stolonifera* and *A*. *scabra*, respectively, due to heat stress.H_2_O_2_ content did not differ between the species under non-stress conditions whereas it remained significantly lower (41% decrease) in *A*. *scabra* compared to *A*. *stolonifera* following heat stress treatment. Histochemical staining for O_2_^-^ and H_2_O_2_ (Fig 2C and 2D, respectively) visually depicted decreased staining intensity for both ROS in *A*. *scabra* roots relative to *A*. *stolonifera* roots following heat stress.

### Non-enzymatic antioxidant content and antioxidant enzyme activities for differential root thermotolerance of *A*. *scabra* and *A*. *stolonifera*

**Fig 3:**
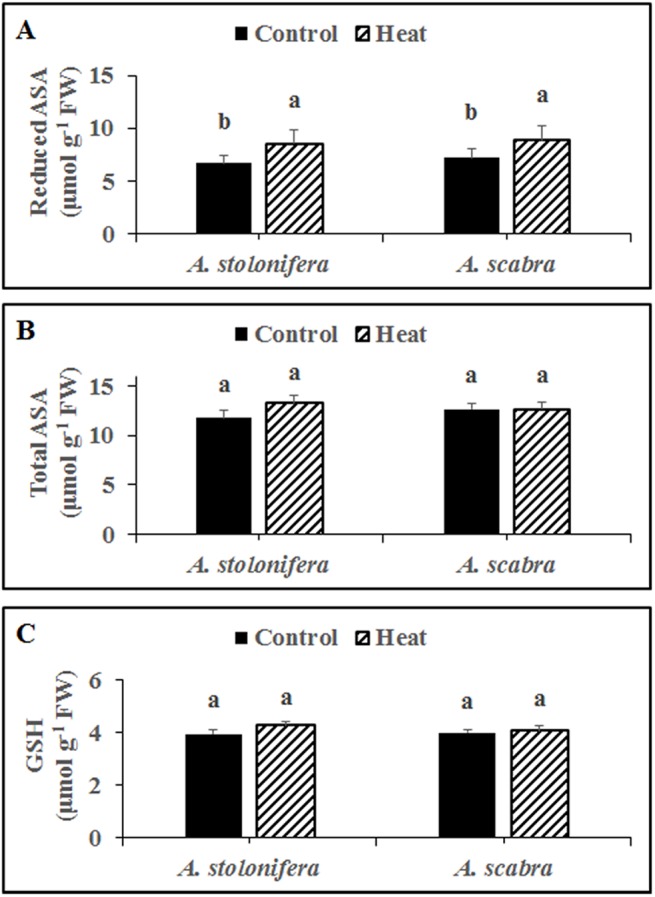
Reduced ascorbate (A), total ascorbate (B) and glutathione (C) content in *A*. *stolonifera* and *A*. *scabra* roots under control and heat stress condition. Data shown are the mean ± SE of four biological replicates. Different letters atop bars indicate significant differences exist at the P ≤ 0.05 level.

Heat stress treatment significantly increased reduced ASA content for both *A*. *stolonifera* (28%) and *A*. *scabra* (23%), compared to respective non-stress controls (Fig 3A). However, there was no difference in reduced ASA content between *A*. *scabra* and *A*. *stolonifera* under heat or non-stress conditions. There was no significant change in total ASA or GSH content for either species due to heat stress compared to respective non-stress controls (Fig 3B and 3C, respectively). Additionally, there was no difference in total ASA or GSH content between *A*. *scabra* and *A*. *stolonifera* under heat or non-stress conditions.

**Fig 4:**
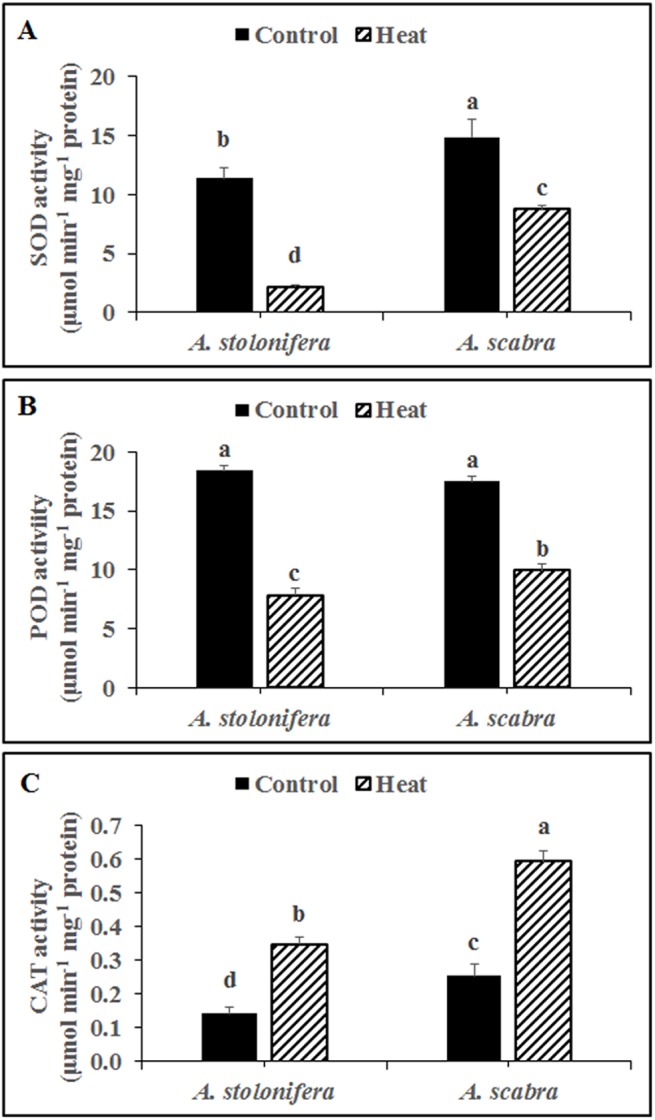
Enzymatic activity of SOD (A), POD (B) and CAT (C) in roots of control or heat-stressed *A*. *stolonifera* and *A*. *scabra*. Data shown are the mean ± SE of four biological replicates. Different letters atop bars indicate significant differences exist at the P ≤ 0.05 level.

Heat stress treatment significantly decreased SOD activity for both grass species compared to respective non-stress controls (Fig 4A). SOD activity decreased by 81 and 41% for *A*. *stolonifera* and *A*. *scabra*, respectively, due to heat stress. SOD activity was significantly higher (31% increase) in *A*. *scabra* compared to *A*. *stolonifera* under non-stress conditions and remained significantly higher (2.98 fold) in *A*. *scabra* compared to *A*. *stolonifera* following heat stress treatment. Heat stress treatment significantly decreased POD activity for both grass species compared to respective non-stress controls (Fig 4B). POD activity decreased by 57 and 43% for *A*. *stolonifera* and *A*. *scabra*, respectively, due to heat stress. There was no difference in POD activity between *A*. *scabra* and *A*. *stolonifera* under non-stress conditions whereas POD activity was significantly higher (27% increase) in *A*. *scabra* compared to *A*. *stolonifera* following heat stress treatment. Heat stress treatment significantly increased CAT activity for both grass species compared to respective non-stress controls (Fig 4C). CAT activity increased 2.5 and 2.4-fold for *A*. *stolonifera* and *A*. *scabra*, respectively, due to heat stress. CAT activity was significantly higher (80% increase) in *A*. *scabra* compared to *A*. *stolonifera* under non-stress conditions and remained significantly higher (70% increase) in *A*. *scabra* compared to *A*. *stolonifera* following heat stress treatment.

**Fig 5:**
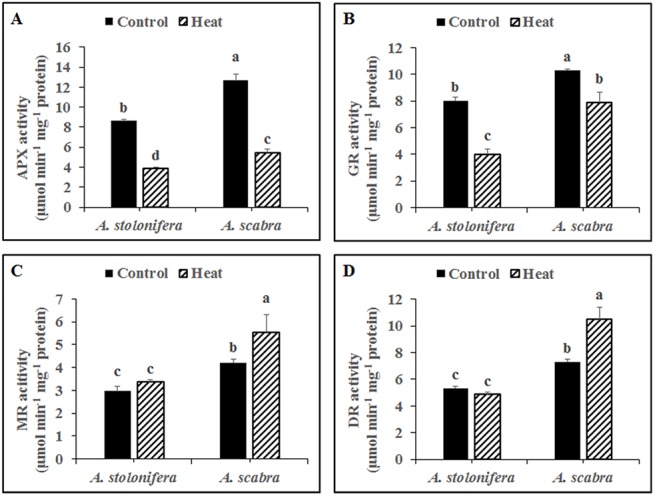
Enzymatic activity of APX (A), GR (B), MR (C) and DR (D) in roots of *A*. *stolonifera* and *A*. *scabra* under control or heat stress condition. Data shown are the mean ± SE of four biological replicates. Different letters atop bars indicate significant differences exist at the P ≤ 0.05 level.

Heat stress treatment significantly decreased APX activity for both grass species compared to respective non-stress controls (Fig 5A). APX activity decreased by 55 and 58% for *A*. *stolonifera* and *A*. *scabra*, respectively, due to heat stress. APX activity was significantly higher (47% increase) in *A*. *scabra* compared to *A*. *stolonifera* under non-stress conditions and remained significantly higher (41% increase) in *A*. *scabra* compared to *A*. *stolonifera* following heat stress treatment. Heat stress treatment significantly decreased GR activity for both grass species compared to respective non-stress controls (Fig 5B). GR activity decreased by 50 and 23% for *A*. *stolonifera* and *A*. *scabra*, respectively, due to heat stress. GR activity was significantly higher (29% increase) in *A*. *scabra* compared to *A*. *stolonifera* under non-stress conditions and remained significantly higher (97% increase) in *A*. *scabra* compared to *A*. *stolonifera* following heat stress treatment. Heat stress treatment significantly increased MR activity for *A*. *scabra* but not for *A*. *stolonifera* compared to respective non-stress controls (Fig 5C). MR activity increased by 32% for *A*. *scabra* due to heat stress. MR activity was significantly higher (41% increase) in *A*. *scabra* compared to *A*. *stolonifera* under non-stress conditions and remained significantly higher (65% increase) in *A*. *scabra* compared to *A*. *stolonifera* following heat stress treatment. Heat stress treatment significantly increased DR activity for *A*. *scabra* but not for *A*. *stolonifera* compared to respective non-stress controls (Fig 5D). DR activity increased by 43% for *A*. *scabra* due to heat stress. DR activity was significantly higher (37% increase) in *A*. *scabra* compared to *A*. *stolonifera* under non-stress conditions and remained significantly higher (116% increase) in *A*. *scabra* compared to *A*. *stolonifera* following heat stress treatment.

### Enzyme gene expression for differential root thermotolerance of *A*. *scabra* and *A*. *stolonifera*

**Fig 6:**
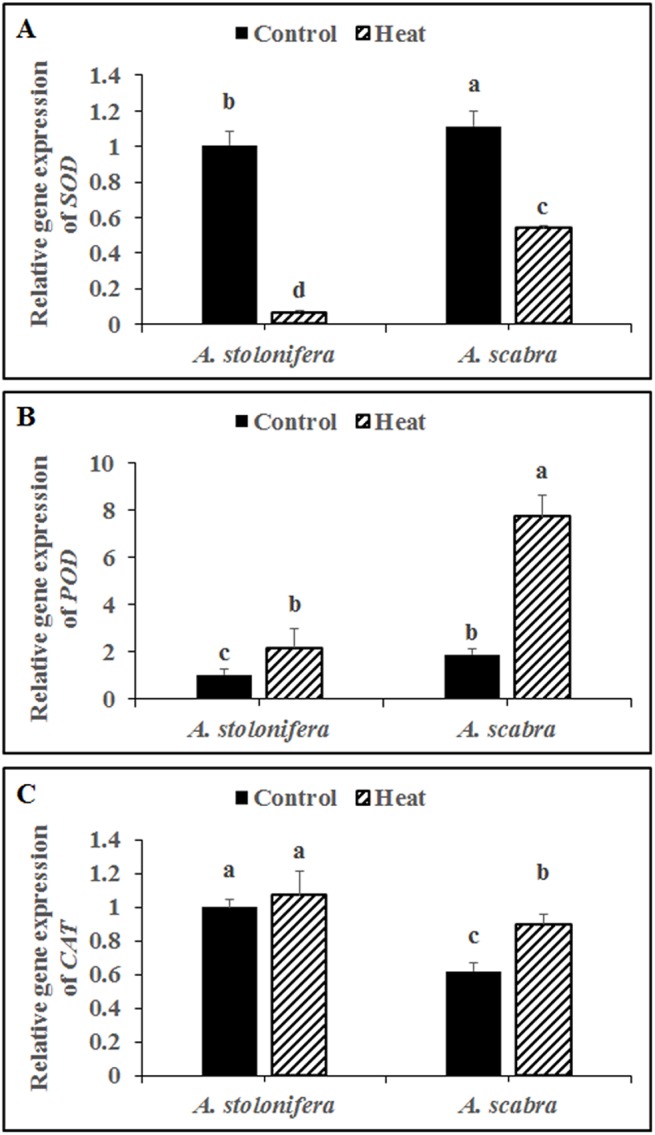
Transcript level of *SOD* (A), *POD* (B) and *CAT* (C) in roots of control or heat-stressed *A*. *stolonifera* and *A*. *scabra*. Data shown are the mean ± SE of four biological replicates. Different letters atop bars indicate significant differences exist at the P ≤ 0.05 level.

Antioxidant enzyme gene transcript levels quantified by qRT-PCR exhibited significant differences between *A*. *scabra* and *A*. *stolonifera* in response to heat stress treatment. Heat stress treatment significantly down-regulated SOD expression for both grass species compared to respective non-stress controls (Fig 6A). SOD expression decreased by 93 and 51% for *A*. *stolonifera* and *A*. *scabra*, respectively, due to heat stress. SOD expression was significantly higher (11% increase) in *A*. *scabra* compared to *A*. *stolonifera* under non-stress conditions and remained significantly higher (6.93 fold) in *A*. *scabra* compared to *A*. *stolonifera* following heat stress treatment. Heat stress treatment significantly up-regulated POD expression for both grass species compared to respective non-stress controls (Fig 6B). POD expression increased 2.1 and 4.1-fold for *A*. *stolonifera* and *A*. *scabra*, respectively, due to heat stress. POD expression was significantly higher (83% increase) in *A*. *scabra* compared to *A*. *stolonifera* under non-stress conditions and remained significantly higher (2.55 fold) in *A*. *scabra* compared to *A*. *stolonifera* following heat stress treatment. Heat stress treatment significantly up-regulated CAT expression for *A*. *scabra* but not for *A*. *stolonifera* compared to respective non-stress controls (Fig 6C). CAT expression increased by 45% for *A*. *scabra* in response to heat stress. CAT expression was significantly lower (38% decrease) in *A*. *scabra* compared to *A*. *stolonifera* under non-stress conditions and remained significantly lower (16% decrease) in *A*. *scabra* compared to *A*. *stolonifera* following heat stress treatment.

**Fig 7:**
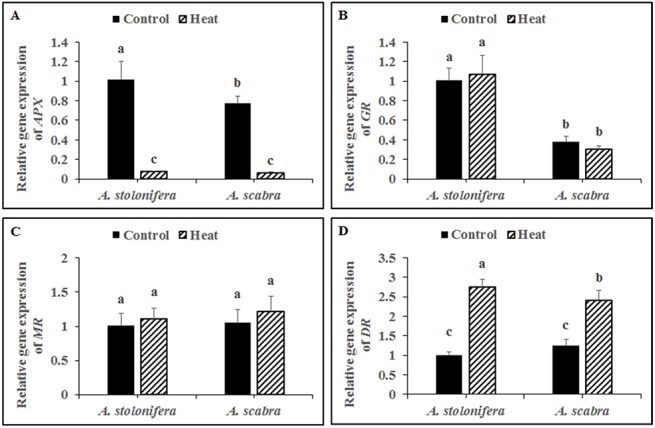
Transcript levels of *APX* (A), *GR* (B), *MR* (C) and *DR* (D) in roots of *A*. *stolonifera* and *A*. *scabra* under control or heat stress condition. Data shown are the mean ± SE of four biological replicates. Different letters atop bars indicate significant differences exist at the P ≤ 0.05 level.

Heat stress treatment significantly down-regulated APX expression for both grass species compared to respective non-stress controls (Fig 7A). APX expression decreased by 92 and 92% for *A*. *stolonifera* and *A*. *scabra*, respectively, due to heat stress. APX expression was significantly lower (24% decrease) in *A*. *scabra* compared to *A*. *stolonifera* under non-stress conditions whereas no significant differences existed between *A*. *scabra* and *A*. *stolonifera* following heat stress treatment. There were no significant differences in GR or MR expression levels for either grass species compared to respective non-stress controls (Fig 7B and 7C, respectively). GR expression was significantly lower (62% decrease) in *A*. *scabra* compared to *A*. *stolonifera* under non-stress conditions and remained significantly lower (72% decrease) in *A*. *scabra* compared to *A*. *stolonifera* following heat stress treatment. There was no difference in MR expression between *A*. *scabra* and *A*. *stolonifera* under heat or non-stress conditions. Heat stress treatment significantly up-regulated DR expression for both grass species compared to respective non-stress controls (Fig 7D). DR expression increased 2.75 and 1.94-fold for *A*. *stolonifera* and *A*. *scabra*, respectively, due to heat stress. DR expression was significantly lower (12% decrease) in *A*. *scabra* compared to *A*. *stolonifera* under heat stress conditions whereas no significant differences existed between *A*. *scabra* and *A*. *stolonifera* under non-stress conditions.

### SNP and SHAM effects on root respiration and ROS production for differential root thermotolerance of *A*. *scabra* and *A*. *stolonifera*

**Fig 8:**
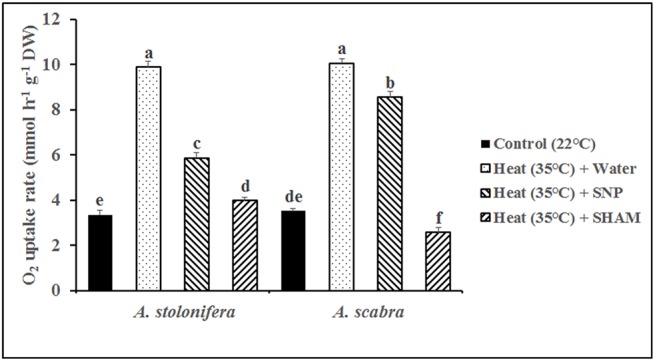
Root respiration rate for *A*. *stolonifera* and *A*. *scabra* under heat stress condition as affected by SNP or SHAM. Data shown are the mean ± SE of four biological replicates. Different letters atop bars indicate significant differences exist at the P ≤ 0.05 level.

**Fig 9:**
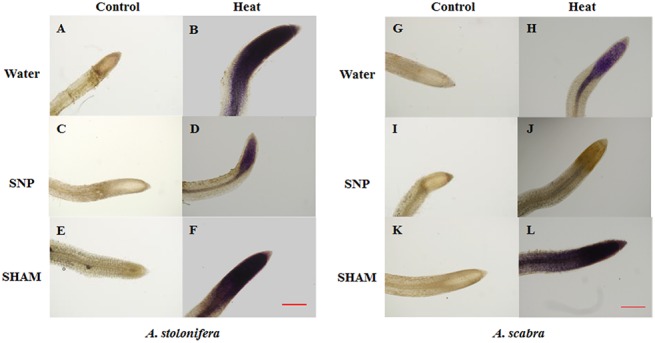
Histochemical staining of *A*. *stolonifera* (A to F) and *A*. *scabra* (G to L) root tips under control and heat stress conditions using NBT. Bar represents 100 μm.

**Fig 10:**
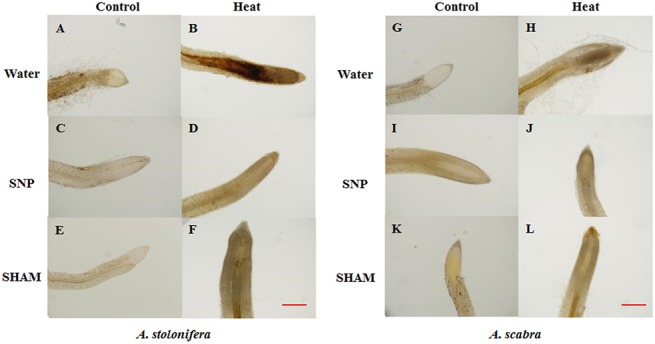
Histochemical staining of *A*. *stolonifera* (A to F) and *A*. *scabra* (G to L) root tips under control and heat stress conditions using DAB. Bar represents 100 μm.

Incubating *A*. *scabra* or *A*. *stolonifera* roots in solutions containing SNP or SHAM revealed significant differences in cytochrome (SHAM-resistant, SNP-inhibited) and alternative (SNP-resistant, SHAM-inhibited) respiration rates responding to heat stress. Heat stress treatment significantly increased total root respiration for both grass species compared to respective non-stress controls (Fig 8). The addition of SNP to incubation solutions reduced respiration rates by 15 and 41% in *A*. *scabra* and *A*. *stolonifera*, respectively, compared to heat-stressed roots with water only. Alternatively, the addition of SHAM to incubation solutions reduced respiration rates by 74 and 60% in *A*. *scabra* and *A*. *stolonifera*, respectively, compared to heat-stressed roots with water only. Furthermore, *A*. *scabra* root respiration rates were significantly lower than non-stressed roots incubated in water only. The results suggest that the alternative respiratory pathway was more involved in the respiratory activities for root tissues of *A*. *scabra* than that of *A*. *stolonifera* under heat stress. Histochemical staining for O_2_^-^ and H_2_O_2_ in SNP and SHAM-incubated roots demonstrated variable accumulation of ROS resulting from changes in cytochrome or alternative respiration rates. *A*. *stolonifera* roots with increased cytochrome respiratory rates and less-active alternative respiration visually depicted an increased staining intensity by NBT for O_2_^-^ (Fig 9A to 9L) and DAB for H_2_O_2_ (Fig 10A to 10L) as compared to *A*. *scabra* roots.

## Discussion

It has been widely recognized that there exists large genetic variability in how roots of different plant species tolerate prolonged period of heat stress, though the contributing underlying mechanisms are not well understood [4]. The current study performed comparative analysis for roots of heat-tolerant *A*. *scabra* and heat-sensitive *A*. *stolonifera* to better describe specific mechanisms contributing to root thermotolerance in cool-season grasses. The commonly-used indicators of cellular membrane stability and integrity (EL and MDA, respectively) showed that *A*. *scabra* roots sustained less membrane damage compared to *A*. *stolonifera* following heat stress treatment. Changes in antioxidant activities and respiratory patterns may prevent or mitigate ROS accumulation to protect cellular membranes and enhance plant root thermotolerance, as discussed below.

Heat-induced ROS formation is a major factor contributing to cellular damages throughout various tissues and organs of many plant species [49]. ROS production results from excessive reductions occurring during respiratory electron transport inducing oxidative damage to nucleic acids, lipids, and proteins [7, 9, 50]. Heat-tolerant *A*. *scabra* accumulated significantly less H_2_O_2_ and O_2_^-^ in roots whereas ROS content was significantly higher in *A*. *stolonifera* roots following heat stress treatment of the current study. The ability of *A*. *scabra* to avoid or minimize H_2_O_2_ and O_2_^-^accumulation in roots may be dependent upon enhanced antioxidant capabilities and/or utilization of efficient respiratory pathways limiting ROS during periods of heat stress.

The plant antioxidant defense system is composed of non-enzymatic (ASA and GSH) and enzymatic (SOD, APX, CAT, POD, GR, MR, and DR) components which effectively scavenge and disable ROS throughout the plant [3]. Root ASA and GSH content did not differ between *A*. *scabra* and *A*. *stolonifera* responding to heat stress suggesting that non-enzymatic antioxidants were either ineffective or not utilized to rid the roots of ROS during heat stress treatment of the current study. Root SOD, POD, CAT, APX, GR, MR, and DR enzyme activities as well as SOD and POD transcript abundances were significantly higher in *A*. *scabra* compared to *A*. *stolonifera* following heat stress. However, APX, GR, MR, and DR activities showed opposite trends compared to the respective transcript levels for each enzyme which may result from significant post translation modification [51]. Another important reason for this discrepancy is the lack of gene family information in these two species. The current knowledge of ROS scavenging-related genes are from previous reports of EST database, which contains incomplete information of Agrostis stolonifera genome [52]. In fact, there is no report on the classification and dissection of ROS scavenging enzyme transcripts in Agrostis spp., which calls for high-throughput sequencing and annotation efforts in future. Nevertheless, the results suggest that enhanced antioxidant activity, particularly for SOD, POD, and CAT, serve important roles for ROS scavenging and cellular maintenance in heat-tolerant *A*. *scabra* roots whereas non-enzymatic components do not.

In addition to the carbon metabolism and energy production needed to support a myriad of plant growth functions, respiratory metabolism may also serve as a major ROS source if left unchecked by various regulatory pathways [9, 53]. Abiotic stressors such as heat, drought, and salinity may increase plant respiration rates and contribute largely towards increased ROS production and accumulation [54–56]. The alterative respiratory pathway involves alternative oxidase (AOX) accepting electrons from ubiquinone and reducing oxygen to water preventing over-reduction of accumulated ubiquinone when cytochrome respiration is otherwise inhibited or restricted by stress [57–59]. Root cytochrome and alternative respiration rates increased for both grass species responding to heat stress in the current study. Incubation in SNP or SHAM to specifically inhibit cytochrome or alternative respiration, respectively, revealed that *A*. *scabra* roots maintained lower cytochrome and higher alternative respiration rates while *A*. *stolonifera* had higher cytochrome and lower alternative respiration rates following heat stress treatment. Minimizing the ROS-producing cytochrome respiratory pathway may contribute to the differential root thermotolerance observed between *A*. *scabra* and *A*. *stolonifera* [60]. Histochemical staining of roots treated with SNP or SHAM further confirmed that ROS were reduced when cytochrome respiration was suppressed in *Agrostis* spp. responding to heat stress treatment.

Respiration inhibitors, such as SNP and SHAM, have been utilized to study the relationship between cytochrome and alternative respiratory pathways in other plant species [61, 62]and have also been used to study the role of alternative respiration in NO signaling [63, 64]. The cytochrome respiration activity in isolated soybean mitochondria was inhibited by the addition of NO solution, while alternative respiration activity was not [30]. *Arabidopsis thaliana* cell cultures incubated with NO donor had increased AOX1a expression and alternative respiration rates [65]. Whether or not AOX expression and alternative respiration are stimulated in cool-season grasses treated with NO deserves further attention in future research. Nevertheless, the results suggest that the collective effects of alternative respiration limitingH_2_O_2_ and O_2_^-^ production rates plus enzymatic antioxidants significantly detoxifying ROS contribute to superior root thermotolerance in *A*. *scabra*.

In summary, *A*. *scabra* roots exhibited superior heat tolerance compared to *A*. *stolonifera*, as demonstrated through physiological and biochemical analysis. The superior root thermotolerance may be due to a highly-efficient enzymatic antioxidant defense system detoxifying ROS from plant roots and active alternative respiration suppressing ROS production. Identifying and characterization of the specific mechanisms of antioxidant scavenging systems is of great benefits for future heat-screening efforts for developing heat-tolerant temperate grass species through genetic modification or molecular breeding.

## Abbreviations

AOX: alternative oxidase
APX: ascorbate peroxidase
ASA: ascorbate
CAT: catalase
DAB: 3-diaminobenzinidine
DR: dehydroascorbate reductase
DTNB: dithiobis-2-nitrobenzoic acid
DTT: dithiothreitol
EDTA: ethylenediaminetetraacetic acid
EL: electrolyte leakage
GR: glutathione reductase
GSH: glutathione
H_2_O_2_: hydrogen peroxide
MDA: malondialdehyde
MR: monodehydroascorbate reductase
NBT: nitroblue tetrozolium
NEM: N-ethylmaleimide
NO: nitrogen oxide
O^2-^: superoxide
PAR: photosynthetically active radiation
POD: peroxidase
PVP: polyvinylpyrrolidone
qRT-PCR: quantitative reverse transcriptase polymerase chain reaction
RH: relative humidity
ROS: reactive oxygen species
SHAM: salicylhydroxamic acid
SNP: sodium nitroprusside
SOD: superoxide dismutase
TBA: thiobarbituric acid
TCA: trichloroacetic acid
XTT: 3-bis(2-methoxy-4-nitro-5-sulfophenyl)-2H-tetrozolium-5-carboxanilide inner salt
